# Arbuscular-Mycorrhizal Networks Inhibit *Eucalyptus tetrodonta* Seedlings in Rain Forest Soil Microcosms

**DOI:** 10.1371/journal.pone.0057716

**Published:** 2013-02-27

**Authors:** David P. Janos, John Scott, Catalina Aristizábal, David M. J. S. Bowman

**Affiliations:** 1 Department of Biology, University of Miami, Coral Gables, Florida, United States of America; 2 Research Institute for the Environment and Livelihoods, Charles Darwin University, Darwin, Northern Territory, Australia; 3 School of Plant Science, The University of Tasmania, Hobart, Tasmania, Australia; Jyväskylä University, Finland

## Abstract

*Eucalyptus tetrodonta*, a co-dominant tree species of tropical, northern Australian savannas, does not invade adjacent monsoon rain forest unless the forest is burnt intensely. Such facilitation by fire of seedling establishment is known as the "ashbed effect." Because the ashbed effect might involve disruption of common mycorrhizal networks, we hypothesized that in the absence of fire, intact rain forest arbuscular mycorrhizal (AM) networks inhibit *E. tetrodonta* seedlings. Although arbuscular mycorrhizas predominate in the rain forest, common tree species of the northern Australian savannas (including adult *E. tetrodonta*) host ectomycorrhizas. To test our hypothesis, we grew *E. tetrodonta* and *Ceiba pentandra* (an AM-responsive species used to confirm treatments) separately in microcosms of ambient or methyl-bromide fumigated rain forest soil with or without severing potential mycorrhizal fungus connections to an AM nurse plant, *Litsea glutinosa*. As expected, *C. pentandra* formed mycorrhizas in all treatments but had the most root colonization and grew fastest in ambient soil. *E. tetrodonta* seedlings also formed AM in all treatments, but severing hyphae in fumigated soil produced the least colonization and the best growth. Three of ten *E. tetrodonta* seedlings in ambient soil with intact network hyphae died. Because foliar chlorosis was symptomatic of iron deficiency, after 130 days we began to fertilize half the *E. tetrodonta* seedlings in ambient soil with an iron solution. Iron fertilization completely remedied chlorosis and stimulated leaf growth. Our microcosm results suggest that in intact rain forest, common AM networks mediate belowground competition and AM fungi may exacerbate iron deficiency, thereby enhancing resistance to *E. tetrodonta* invasion. Common AM networks–previously unrecognized as contributors to the ashbed effect–probably help to maintain the rain forest–savanna boundary.

## Introduction

Eucalypts predominate across much of monsoon tropical, northern Australia. *Eucalyptus tetrodonta* F.Muell. and *E. miniata* Cunn. ex Schauer are canopy co-dominants of coastal savannas [1] throughout which are scattered patches of rain forest [2], [3]. The savannas are highly susceptible to fire, but only after very dry conditions can fires cross the abrupt ecotone between savanna and rain forest [4]. If high-intensity fires penetrate the rain forest, then *E. tetrodonta* seedlings can invade, but otherwise they cannot, even after canopy destruction by tropical cyclones [5]. Reciprocally, in the absence of fire, rain forest plants can colonize savanna, albeit very slowly [6]. Nevertheless, the mechanisms by which fire facilitates rain forest invasion by *E. tetrodonta* are uncertain.

The apparent necessity of fire to facilitate establishment of some species' seedlings, especially those of eucalypts and pines, is known as the "ashbed effect" (a tenet which underpins much Australian forestry practice [7]). Although the ashbed effect has been investigated for more than half a century, its mechanisms remain ambiguous because it likely involves multiple phenomena associated with fire and soil desiccation. Those phenomena may include at different times and places, direct fertilization by ash [7], soil physical and chemical changes that diminish P adsorption [7], [8], release of mineral nutrients from heat-killed soil microorganisms [8] (but see [9]), partial soil sterilization that eliminates pathogenic microbes [10] (but see [11]), or other alterations of the soil microflora, especially ectomycorrhizal fungi [12], [13]. Notwithstanding uncertainty about the mechanisms behind the ashbed effect, empirical evidence from across Australia shows that without fire, rain forest resists invasion by savanna plant species, just as fire contributes to savannas' resistance to replacement by rain forest [4].

**Figure 1:**
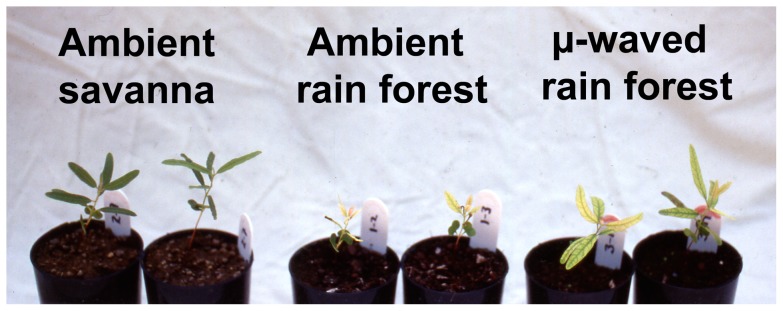
*Eucalyptus tetrodonta* seedlings in ambient savanna, ambient rain forest, and microwaved rain forest soil. The seedlings were transplanted 58 d previously to this pilot experiment (data not shown). True leaves of the seedlings in their native savanna soil are dark green, but those of seedlings growing in ambient rain forest soil are chlorotic and extremely stunted. Seedlings in microwave-heated (µ-waved) rain forest soil are not stunted versus those in savanna soil, but their leaves are chlorotic, exhibiting the dark green veins and yellow inter-vein areas characteristic of iron deficiency. All root systems were sparse, and no mycorrhizas of any type were apparent.

Bowman and Fensham [5] demonstrated that soil fumigation alone can mimic the ashbed effect in facilitating *E. tetrodonta* seedling growth in rain forest soil. Although the success of fumigation suggests a primary role of soil microflora in the ashbed effect, whether that role mainly is attributable to mineral nutrient release from killed saprotrophs, elimination of pathogens and parasites, or changes in mycorrhizal fungi is uncertain. *E. tetrodonta*, *E. miniata*, and other common tree species of the savanna are ectomycorrhizal (ECM) as adults [14], but Australian rain forest tree species almost exclusively comprise arbuscular mycorrhizal (AM) hosts [15]. So, we hypothesized that rain forest AM fungi are detrimental to *E. tetrodonta* seedlings, and that, much like intense burns, soil fumigation or partial sterilization (as in a pilot experiment that we conducted; Figure 1) relieves the detriment. Although fire more often negatively affects ECM than AM fungi [16], [17], intense fires burning into rain forest kill AM host plants and thereby might disrupt otherwise persistent networks of mycorrhizal interconnections among those plants.

Accumulating evidence supports the inference that plants can be interconnected by mycorrhizal fungus hyphal networks, often called "common mycorrhizal networks" [18]–[20]. These constellations of hyphal bridges among root systems have been suggested to be pathways for inter-plant movement of fixed-carbon by both ECM [21] and AM [22] fungi, mineral nutrients such as nitrogen [23], [24] and phosphorus [25], [26] by both types of mycorrhizal fungi, and water, the latter especially by ECM networks [27].

Ectomycorrhizal networks in the savanna might facilitate seedling establishment by redistributing fixed carbon (possibly in the form of nitrogenous compounds [28]), mineral nutrients [20], and water [29]. Additionally, ECM networks may favor seedlings simply by maintaining a high density of fungi that lead to rapid mycorrhiza formation, or by influencing mycorrhizal fungus species composition [20], [30]. Arbuscular mycorrhizal networks, however, are not likely to supply fixed carbon to host plants because transported carbon probably remains as storage lipids within the fungi in receiver plant roots [31]. Moreover, because AM fungi generally do not produce rhizomorphs or mycelial strands, they likely do not redistribute water to the same extent as ECM networks. In the savanna, unless coexisting ECM and AM networks include plant species such as eucalypts that can form both types of mycorrhizas [32] and thereby might link their networks, ECM and AM networks probably represent distinct niches [30].

Arbuscular mycorrhizal fungus networks may be as likely to intensify belowground competition as they are to enhance mycorrhiza formation and overall mineral nutrient acquisition. Although Chiariello et al. [25] found in a short-term, pulse-chase, field experiment with P^32^ that it was distributed to plants surrounding a decapitated donor without relationship to distance to recipient, recipient size, or recipient species, several greenhouse experiments have suggested that plants interconnected by AM networks can compete strongly belowground [33]–[35]. Our hypothesis of AM fungus detriment to *E. tetrodonta* seedlings anticipates that within a rain forest competitive milieu, *E. tetrodonta* seedlings will be disadvantaged by inclusion within a common AM network. Thereby, instead of AM and eventual ECM hosts coexisting relatively benignly as often tacitly assumed, we propose that in some situations they may interact antagonistically.

We designed a microcosm experiment to examine our hypothesis that failure of *E. tetrodonta* seedlings to grow in rain forest soil is a consequence of inhibition by common AM networks. Because *Eucalyptus* species are susceptible to iron deficiency [36] and we observed symptoms suggestive of iron deficiency in rain forest soil in a pilot experiment with *E. tetrodonta* seedlings (Figure 1), as an additional treatment 130 days after transplant (DAT), we applied soluble iron to one-half of the *E. tetrodonta* seedlings in ambient soil to test an additional hypothesis that iron deficiency limited their growth.

## Materials and Methods

### Experiment design

**Figure 2:**
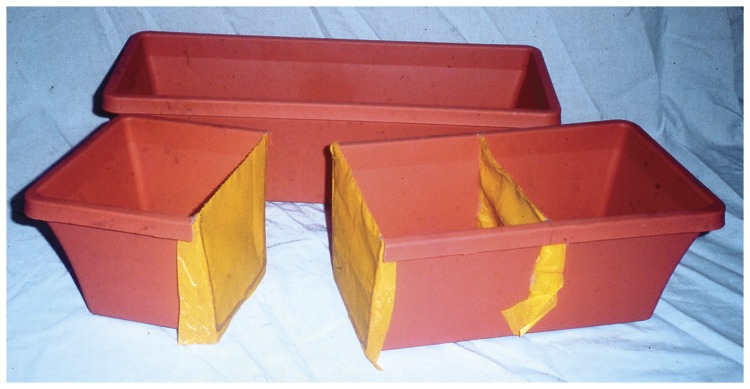
A disassembled microcosm. The three-compartmented planter box is divided by double layers of nylon silk-screen cloth mesh root barriers (45 µm pore size). A double layer was cemented into a slot on the right side, and single layers were cemented across each cut end of the completely separated left side. When assembled, the compartmented box nested tightly within the fully-intact outer box, but the separated end periodically could be lifted vertically, sliding the layers of cloth mesh against one another and severing hyphae that crossed them.

We grew 40 *E. tetrodonta* seedlings (10 in each of four treatments) in the end compartments of rectangular microcosms divided into three equal compartments by nylon mesh root barriers through which AM fungus hyphae could pass (Figure 2). In the central compartments, "nurse" plants in ambient ( = not fumigated) rain forest soil sustained AM fungi. Hyphae could extend from the nurse plant into the two end compartments potentially to establish common mycorrhizal networks with seedlings at both ends either in ambient soil or in soil fumigated initially to eliminate AM fungi. Hyphae extending into one end compartment of each microcosm could be severed repeatedly to disrupt connections to the nurse plant. Thus, there were four treatments in a split-plot, factorial design: ambient or fumigated soil in both end compartments of individual microcosm "plots" crossed with intact (hereafter called "networked") or severed common mycorrhizal networks.

In order to determine if soil fumigation and repeated hypha severing succeeded in diminishing AM formation as intended, we grew 40 seedlings of an AM-responsive [37] tropical tree species, *Ceiba pentandra* (L.) Gaertn. (Malvaceae) in microcosms to which we applied the same four treatments as for *E. tetrodonta*. We did not grow *C. pentandra* together with *E. tetrodonta* in the same microcosms because we did not wish them to compete with one another (although both could compete with their respective nurse plants).

### Establishment of microcosms

We constructed the microcosms from plastic planter boxes (15 cm×45 cm×15 cm deep; Figure 2). Each was divided into thirds, at one side by a slit sawn to, but not through, the rim, and at the opposite side by entirely separating the end one-third. In the slit, we cemented with silicone sealer a double layer of nylon silk-screen cloth mesh (45 µm pore size) that was a barrier to roots but not to AM fungus hyphae or other microorganisms. Similarly, across the fully-cut ends we cemented single pieces of mesh. Thereby, both ends were separated from the central compartment by two layers of mesh. Each modified box was nested within an intact box in which it fit tightly, but which allowed the cut end to be lifted momentarily in order to sever fungus hyphae that crossed the double mesh root barrier. Soil pressed the layers of mesh together tightly so there was no air gap between them.

We filled the microcosm central compartments with rain forest soil (Table 1) collected to no more than 20 cm depth at Gunn Point (12°09.258′ S, 131°02.186′ E) near Darwin, NT, Australia. We filled the two end compartments with either the same ambient soil, or with that soil fumigated with methyl bromide gas at 1 kg m^−3^. As supplemental inoculum of indigenous AM fungi, we collected fine roots from the Gunn Point rain forest one-day prior to use. The roots were hand cut into 1–2 cm pieces and were layered in central compartments at half their depth. Above this layer of fresh roots, we transplanted a 15–20 cm tall, field-collected root sprout of the Australian rain forest tree species *Litsea glutinosa* (Lour.) C. B. Rob. (Lauraceae). Before transplant, each sprout's source root was treated by dipping it into powdered rooting hormone and micronutrients ("Clonex"). Then, the compartments were filled with ambient soil topped with 2–3 cm of fumigated soil to reduce the chance of spores splashing into end compartments. We filled both end compartments of 20 microcosms with ambient soil, and filled those of an additional 20 microcosms with fumigated soil. The end compartments were neither actively inoculated with AM fungi, nor were they treated with a microbial filtrate [38], [39] because the central compartments likely would serve as a source of bacteria and saprotrophic fungi as well as AM fungi.

**Table 1:** Attributes of Australian Northern Territory monsoon rain forest and *Eucalyptus* savanna soils.

Attribute (units)	Rain forest	Savanna	*t*, *P*
pH	**6.3** (0.1)	5.0 (0.3)	5.01, 0.038
EC (mS cm^−1^)	**0.26** (0.03)	0.10 (0.02)	4.44, 0.047
Ca (mg kg^−1^)	**3050** (50)	330 (130)	19.53, 0.003
Mg (mg kg^−1^)	**305** (5)	130 (30)	5.75, 0.029
K (mg kg^−1^)	**145** (5)	27 (3)	20.24, 0.002
Na (mg kg^−1^)	<25 (0)	<25 (0)	NA
P (Colwell; mg kg^−1^)	**23** (1)	8 (1)	13.86, 0.005
S (mg kg^−1^)	39 (5)	37 (5)	0.28, 0.804
TKN (%)	**0.31** (0.04)	0.11 (0.02)	4.80, 0.041
Cu (mg kg^−1^)	0.7 (0.0)	0.6 (0.1)	3.01, 0.204
Fe (DTPA; mg kg^−1^)	17 (1)	**43** (2)	−12.85, 0.006
Mn (mg kg^−1^)	101 (12)	156 (60)	−0.91, 0.460
Zn (mg kg^−1^)	**7.8** (0.5)	1.0 (0.7)	8.60, 0.013
OC (%)	**7.20** (0.46)	1.69 (0.07)	11.96, 0.007
Ex Al (me%)	0.01 (0.00)	0.31 (0.29)	−1.03, 0.489

Values are means ±1 standard error in parentheses with significantly highest values in bold. Soils (n = 2 for each) were compared by *t*-test (approximate *t*-test for unequal variances for Cu and exchangeable Al); *t* statistics and probabilities are shown. EC = electrical conductivity; TKN = total Kjeldahl N; OC = organic carbon; Ex Al = exchangeable Al; NA = not tested (below detection limit).

After transplanting *L. glutinosa* sprouts on 20 June, 1997, we randomized all 40 microcosms with no more than 2 cm separation in two rows on a wire mesh screenhouse bench at the CSIRO Berrimah campus (12°24.800′ S, 130°55.280′ E) in Darwin. The screenhouse provided light shade. The nurse plants grew in the microcosms for six months before we transplanted *E. tetrodonta* and *C. pentandra* seedlings to begin the experiment. This pre-treatment allowed AM fungus hyphae to extend into stationary end compartments, and allowed hyphae from propagules in stationary, ambient-soil end compartments potentially to reach nurse plant roots. In order to disrupt hypha spread, three days after transplanting the *L. glutinosa* sprouts, we began lifting the detached end compartments of each microcosm by 8–10 cm every two or three days. We continued this periodic severing of hyphae until all plants were harvested.

### Seedling growth, measurement, and harvest

Seeds of *E. tetrodonta* obtained from Top End Seeds (Nightcliff, NT) and of *C. pentandra* obtained from the George Brown Botanic Gardens (Darwin) were sown in germination flats of fumigated rain forest soil in the screenhouse. On 20 December, 1997, we transplanted two 1–2 week-old *E. tetrodonta* or three just-emerged *C. pentandra* seedlings into both end compartments of separate microcosms. One week later on 28 December, we replaced *C. pentandra* of which all had died in 22 microcosm ends. A week before the first growth measurement, we clipped the weakest individuals of *C. pentandra* and *E. tetrodonta*, leaving only one seedling in each end compartment. Thus, there were 40 individuals (10 per treatment) in 20 microcosms for each species (40 microcosms in total).

During the rainy season (October–April) all microcosms received natural rainfall and were watered only if necessary, but during the dry season they were watered three times daily by an overhead sprinkler system. Mean monthly rainfall from December, 1997 through April, 1998 ranged from 75 mm to 789 mm, and mean monthly maximum and minimum air temperatures ranged from 31.6 to 33.9 and 24.9 to 25.5 °C, respectively. While we continued to grow *E. tetrodonta* seedlings in ambient soil with or without iron fertilization from May through July, 1998, mean monthly rainfall was less than 0.4 mm, and the maximum and minimum mean monthly temperatures were 33.4 and 21.4 °C, respectively (all weather information for Darwin airport from www.bom.gov.au).

Beginning on 19 January, 1998, one month after transplant, and continuing every two weeks until harvest, we measured height from the soil surface to the shoot apex, length of the longest leaflet or leaf, and counted the number of leaves of every seedling. Additionally, we categorically recorded foliar chlorosis of *E. tetrodonta*. We measured *L. glutinosa* nurse plants only twice, on 22 December, 1997, at the start of the experiment, and on 10 May, 1998. We measured *L. glutinosa* stem diameter, height from the soil surface to the tallest shoot apex, longest leaf length, and counted the number of leaves.

On 10 May, 1998, 141 DAT, we harvested all *C. pentandra* and only the *E. tetrodonta* grown in fumigated soil. We harvested at this time because of declining health and mortality of *E. tetrodonta*, especially in networked, ambient soil. Root systems of both species were extracted from the soil, rinsed gently over a 1 mm sieve, and preserved in 50% ethanol for assessment of mycorrhizal colonization. For *E. tetrodonta* and their accompanying *L. glutinosa* nurse plants, we separated leaf blades from petioles and stems, dried all to constant weight in an oven at 60 °C, and then weighed them.

### Iron fertilization

We did not harvest the *E. tetrodonta* seedlings grown in ambient soil when we harvested the seedlings from fumigated soil. Instead, we continued to grow them for an additional 69 days with or without iron fertilization. We randomly assigned half of each group of hyphae-severed and networked seedlings to receive 81 mg of iron (100 mL of 3.24 g L^−1^ of "Librel Fe–Lo", Allied Colloids, England; water soluble Fe = 13.2% and Fe chelated by EDTA = 12.5%) eight times at irregular intervals (i.e., on 29 April, 13, 21, 28 May, 4, 11, 24 June, and 11 July, 1998). This addition was intended transiently to elevate the available iron concentration of rain forest soil approximately to that of savanna soil (Table 1). We monitored these seedlings' growth, and processed them and their nurse plants at harvest (210 DAT) as described previously.

### Mycorrhizal colonization and leaf tissue analyses

Preserved root systems of all *E. tetrodonta* seedlings were blotted dry and weighed before haphazardly removing a fine-root sample for assessment of mycorrhizal colonization. Afterwards, the remaining roots were weighed again before being dried to constant weight. We used dry-weight to fresh-weight ratios to calculate the dry weights of entire root systems.

For both *C. pentandra* and *E. tetrodonta*, we cleared fine-root samples in 10% KOH at room temperature for 48 h before acidifying them in 5% HCl for 15 min, and then stained them in 0.05% trypan blue in lactoglycerol at room temperature for 6 hr. We mounted ten to twenty, 1–2 cm long root segments on microscope slides and scored them for percentage length colonized by AM fungi (typical hyphae and vesicles in the root cortex) with a compound microscope according to the magnified gridline intersection method [40]. We usually examined 160 to more than 500 intersections per plant, but examined fewer than 100 intersections for seven *E. tetrodonta* with very small root systems. We excluded colonization data of two small individuals from the fumigated soil, networked treatment because they were outliers (below the lower 99.9% confidence limit for the other plants of that treatment).

*E. tetrodonta* leaf tissues (petioles excluded) were analyzed for element concentrations by the Department of Primary Industries & Fisheries, Berrimah Agricultural Research Centre, Chemistry Section (Berrimah). Leaf tissues of as many individuals as possible were analyzed separately, but because some *E. tetrodonta* had tiny leaves, we composited tissue of 2–4 plants as necessary. There was one composite of 2 fumigated soil, hyphae-severed plants; three composites (two of 2 and one of 3 plants) of fumigated soil, networked plants; one composite of 4 ambient soil, hyphae-severed plants; and two composites of 2 ambient soil, networked plants. The Chemistry Section also analyzed ambient and fumigated composited samples of the rain forest soil, and for comparison, ambient and fumigated samples of savanna soil collected 0.57 km distant from the rain forest. Soil pH and electrical conductivity were determined in a 1:5 soil:water extract; K, Ca, Mg, and Na in ammonium chloride; Colwell P in sodium bicarbonate; S in calcium dihydrogen phosphate; Zn, Cu, Mn, and Fe in DTPA; organic carbon by modified Walkley-Black digestion; and exchangeable Al in calcium chloride.

### Statistical analyses

We used *t*-tests (approximate *t*-tests for Cu and exchangeable Al) to compare rain forest and savanna soil (n = 2 for each soil), and we report as significantly different (Table 1) those attributes for which *P*≤0.05. All statistical tests except those noted otherwise were performed with Statistix v. 9.0 (Analytical Software, Tallahassee, FL).

We analyzed *C. pentandra* percentage root length colonized by AM fungi by split-plot, two-factor ANOVA with soil fumigation as the whole-plot factor, but because of different harvest dates, we analyzed *E. tetrodonta* AM colonization by separate two-sample *t*-tests for each harvest. Percentage colonized root length was arcsine-square root transformed before analysis. *E. tetrodonta* dry weight variables were compared by paired *t*-tests (pairing plants in the same microcosm) for plants in fumigated soil, and by *t*-tests (because of unequal numbers of surviving plants) for plants in ambient soil after Bonferroni correction for the number of analyzed response variables (*P*≤0.0125 = 0.05/4 dry weight response variables).

We analyzed morphological responses of *C. pentandra* and *E. tetrodonta* by split-plot, two-factor, repeated-measures ANOVAs after Levene's test of heteroscedasticity. Applying a Bonferroni correction for three morphometric response variables, we report as significant, effects for which *P*≤0.017. Mortality of *E. tetrodonta* in the ambient, networked treatment unbalanced the numbers of seedlings per treatment, so we used JMP Pro v. 10.0.0 (SAS Institute, Inc., Cary, NC) with restricted maximum-likelihood calculations to perform the *E. tetrodonta* repeated-measures analyses.

We used a one-way MANOVA performed with JMP Pro v. 10.0.0 followed by univariate analyses of the four *L. glutinosa* response variables to compare the effects on *L. glutinosa* of being in a microcosm with *C. pentandra* versus being with *E. tetrodonta*. Because *L. glutinosa* individuals were 15–20 cm tall at transplant, we relativized final morphological measurements by using their differences from initial measurements. We examined hypothesized negative associations of *C. pentandra* and *E. tetrodonta* with *L. glutinosa* for effects of severing common mycorrhizal networks with Pearson's correlations. Because we did not determine the dry weights of *C. pentandra*, we analyzed relativized final morphological measurements for *C. pentandra* (longest leaflet length change) versus *L. glutinosa* number of leaves. For *E. tetrodonta* versus *L. glutinosa*, however, we analyzed shoot dry weights. One ambient, networked *E. tetrodonta* seedling with a dry weight (1.67 g) almost twice the upper 99.9% confidence limit for its treatment was excluded.

We analyzed morphological responses of *E. tetrodonta* to iron fertilization over 69 d following the initial fertilization by repeated-measures ANOVAs performed with JMP Pro v. 10.0.0, followed by Bonferroni correction (*P*≤0.017) for having examined three response variables. We used Fisher exact tests to examine the effects of iron fertilization on recovery from chlorosis.

We analyzed element concentrations in *E. tetrodonta* foliage across both harvests by one-way ANOVA or non-parametric Kruskal-Wallis tests if variances were heteroscedastic. We used a Bonferroni-corrected P0.0045 (=0.05/11 elements) to assess the significance of these ANOVAs and of Pearson's correlation coefficients between *E. tetrodonta* leaf dry weights and element concentrations.

## Results

### Soil attributes

Among the 15 soil attributes shown in Table 1, ten differed significantly, but only one, iron, was greater in savanna soil than in rain forest soil. Those mineral nutrient concentrations that differed significantly were 2.3 (Mg) to 9.2 (Ca) times higher in rain forest than in savanna soil, except for available iron which was 2.6 times more abundant in savanna than in rain forest soil.

### *C. pentandra* mycorrhizas and growth

**Figure 3:**
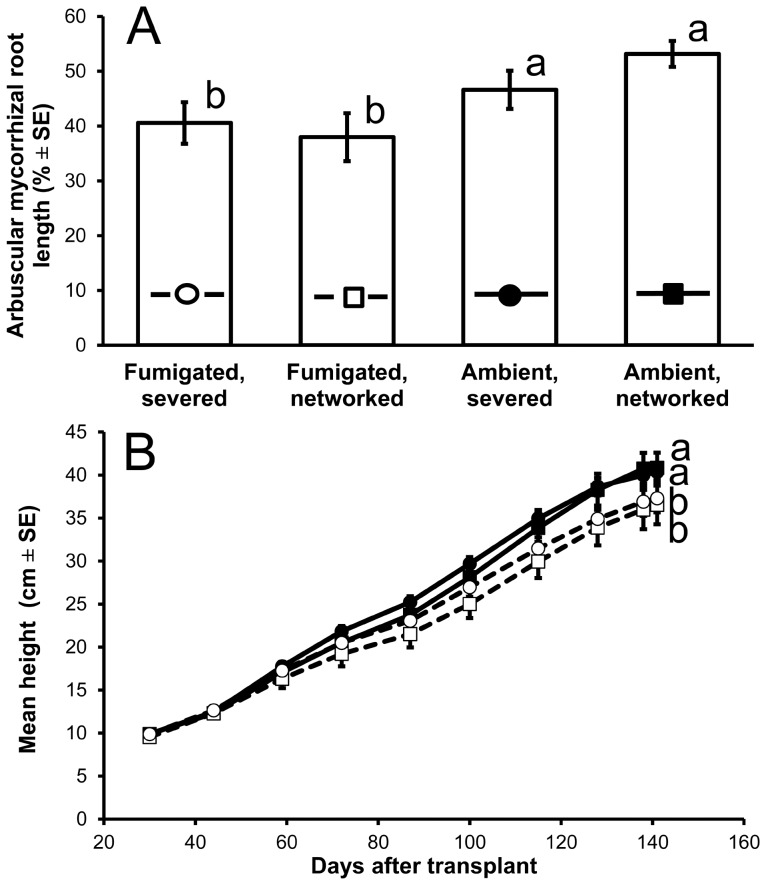
*Ceiba pentandra* arbuscular mycorrhizas and height growth responses. (A) arbuscular mycorrhizal root length (%±1 standard error) at 141 days after transplant, and (B) mean height (cm±1 standard error) versus days after transplant to microcosms with *Litsea glutinosa* AM nurse plants containing ambient (filled symbols; solid lines) or methyl-bromide fumigated (open symbols; dashed lines) rain forest soil in which hyphae repeatedly were severed (circles) or not (squares). Symbols on bars in Figure 3A correspond to those in Figure 3B. Bars topped by the same lowercase letter in 3A do not differ significantly; in 3B, letters similarly denote interactions with time. Fumigation reduced colonized root length and diminished mean height increase. (Neither hypha severing nor its interaction with fumigation significantly affected either root colonization or growth.)

Fumigation significantly reduced *C. pentandra* AM-colonized root length (*F*_1,9_ = 7.13, *P* = 0.026) but colonization was not affected significantly by severing hyphae (*F*_1,18_ = 0.00, *P* = 0.957) or by the interaction of fumigation and severing hyphae (*F*_1,18_ = 1.66, *P* = 0.214; Figure 3A). At harvest, 141 DAT, *C. pentandra* seedlings averaged 50% AM-colonized root length in ambient soil, but only 39% in fumigated soil.

Fumigation significantly diminished height increase (fumigation×time *F*_9,162_ = 4.96, *P*<0.001; Figure 3B) and number of leaves increase (fumigation×time *F*_9,162_ = 3.02, *P* = 0.002). At 141 DAT, mean seedling height differed by 3.7 cm and mean number of leaves by 0.7 leaves in fumigated versus ambient soil. Increase of longest leaflet length, which appeared to have reached an asymptote at 8.0 cm, was not significantly affected by soil fumigation (fumigation×time *F*_9,162_ = 1.85, *P* = 0.063). We found no significant effects of severing hyphae on *C. pentandra* growth rates (height: severing×time *F*_9,162_ = 1.05, *P* = 0.399; leaflet length: severing×time *F*_9,162_ = 2.30, *P* = 0.018; number of leaves: severing×time *F*_9,162_ = 1.21, *P* = 0.293), nor did fumigation, severing, and time interact for any morphological response variable (all *P*>0.544).

### *E. tetrodonta* mycorrhizas and seedling performance

**Figure 4:**
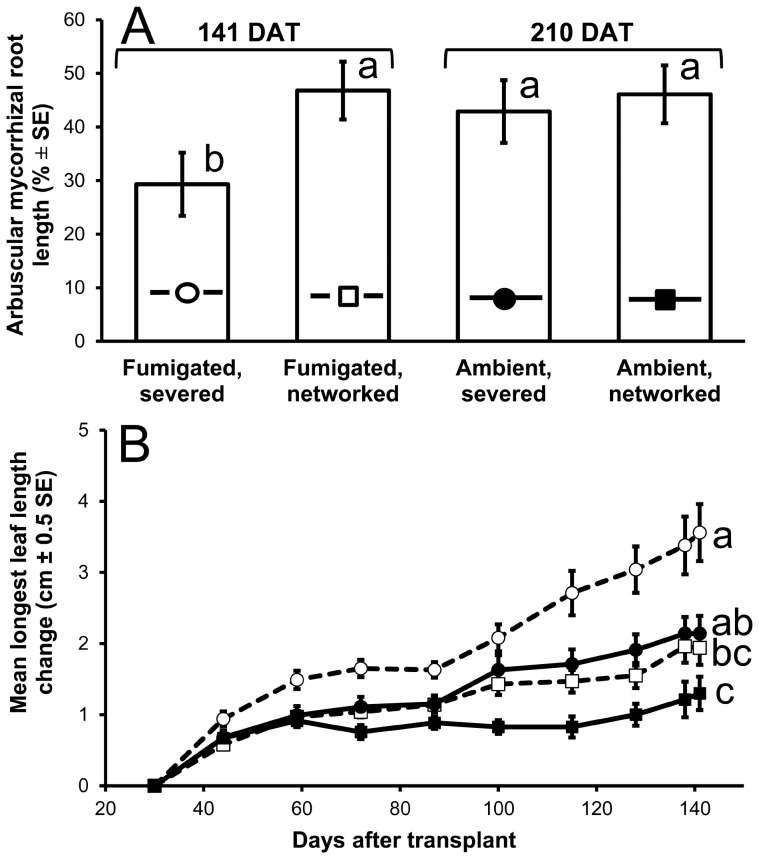
*Eucalyptus tetrodonta* arbuscular mycorrhizas and longest leaf length growth responses. (A) arbuscular mycorrhizal root length (%±1 standard error) at 141 days after transplant (DAT) to methyl-bromide fumigated rain forest soil and at 210 DAT to ambient soil, and (B) mean longest leaf length change from the initial measurement (cm±0.5 standard error) versus days after transplant to microcosms with *Litsea glutinosa* AM nurse plants containing ambient (filled symbols; solid lines) or methyl-bromide fumigated (open symbols; dashed lines) soil in which hyphae repeatedly were severed (circles) or not (squares). Symbols on bars in Figure 4A correspond to those in Figure 4B. Bars topped by the same lowercase letter in 4A do not differ significantly within a harvest; in 4B, letters similarly denote interactions with time. Severing network hyphae reduced colonized root length in fumigated soil, but not in ambient soil. Severing network hyphae increased mean longest leaf length change, but neither fumigation nor its interaction with severing significantly affected longest leaf length change.

Severing common mycorrhizal network hyphae significantly reduced *E. tetrodonta* AM-colonized root length in fumigated soil 141 DAT (df = 16, *t* = 2.15, *P* = 0.047), but in ambient soil 210 DAT, it did not (df = 15, *t* = 0.39, *P* = 0.701; Figure 4A). In ambient soil, 45% of *E. tetrodonta* root length was colonized by AM fungi, which was similar to the 47% colonized root length of seedlings in fumigated soil with intact networks. Hyphae-severed seedlings in fumigated soil had only 29% colonization. We did not find any ectomycorrhizas.

Three of ten *E. tetrodonta* seedlings in ambient soil with intact common mycorrhizal networks failed to survive until 141 DAT. Among surviving seedlings, severing hyphae significantly elevated height increase (severing×time *F*_9,147.7_ = 5.96, P0.001), longest leaf length increase (severing×time *F*_9,150.4_ = 4.53, *P*<0.001; Figure 4B), and number of leaves increase (severing×time *F*_9,146.4_ = 3.59, P0.001). Hyphae-severed seedlings in fumigated soil exceeded the mean height of seedlings in all other treatments by 3.4 cm, the mean longest leaf length by 2.2 cm, and the mean number of leaves by 3.4. We found no significant effects of fumigation on any morphological variable (height: fumigation×time *F*_9,163.8_ = 0.26, *P* = 0.985; leaf length: fumigation×time *F*_9,158.3_ = 1.82, *P* = 0.069; number of leaves: fumigation×time *F*_9,161.7_ = 0.47, *P* = 0.895), nor did fumigation, hyphae severing, and time interact significantly for any variable (all *P*>0.456).

Leaf and stem dry weights of *E. tetrodonta* seedlings grown in fumigated soil were significantly increased by severing hyphae, but total root dry weights and fine root-to-leaf dry weight ratio did not differ (Table 2). Hyphae-severed seedlings grown an additional 69 days in ambient soil without iron fertilization consistently exceeded the dry weights of networked seedlings, although not significantly (Table S1).

**Table 2:** Dry weight responses of *Eucalyptus tetrodonta* seedlings to common mycorrhizal network hypha severing in fumigated rain forest soil and to soluble iron fertilization in non-fumigated, ambient soil.

	Fumigated soil (141 DAT)			Ambient soil (210 DAT)		
Response (units)	Hyphae severed	Networked	DF, *t*, *P*	No fertilization	Iron fertilization	DF, *t*, *P*
Leaf weight (g)	**0.480** (0.338)	0.202 (0.053)	9, 3.14, 0.012	0.380 (0.118)	0.504 (0.168)	15, 0.61, 0.549
Stem weight (g)	**0.187** (0.033)	0.085 (0.022)	9, 4.05, 0.003	0.175 (0.044)	0.204 (0.058)	15, 0.40, 0.695
Root weight (g)	0.090 (0.032)	0.037 (0.013)	9, 2.02, 0.074	0.208 (0.088)	0.206 (0.082)	15, 0.02, 0.988
Fine roots:leaf	0.084 (0.025)	0.065 (0.012)	9, 0.91, 0.384	0.319 (0.090)	0.181 (0.033)	10.1, 1.44, 0.181

Values are means±1 standard error in parentheses with significantly highest values in bold. Degrees of freedom (DF), *t* statistics, and associated two-tail probabilities (*P*) are shown. Satterthwaite's *t*-test (with fractional degrees of freedom) was used when variances were not homogenous. In methyl-bromide fumigated soil, n = 10 for both groups, but in non-fumigated, ambient soil, 9 plants were not fertilized versus 8 plants fertilized. DAT = days after transplant.

### Common mycorrhizal network effects on plant interactions

*C. pentandra* seedlings were conspicuously larger than *E. tetrodonta* seedlings at harvest. A one-way MANOVA with all four *L. glutinosa* response variables showed that the *L. glutinosa* nurse plants grew significantly less (*F*_4,35 = _2.95, *P* = 0.033) when accompanied by *C. pentandra* than when accompanied by *E. tetrodonta*. Univariate analyses of *L. glutinosa* nurse plant height change (*F*_1,38 = _3.19, *P* = 0.082), largest leaf length change (*F*_1,38 = _4.32, *P* = 0.044), number of leaves change (*F*_1,38 = _9.96, *P*<0.001), and stem diameter change (*F*_1,38 = _6.57, *P* = 0.014) revealed significant effects on the latter three response variables.

**Figure 5:**
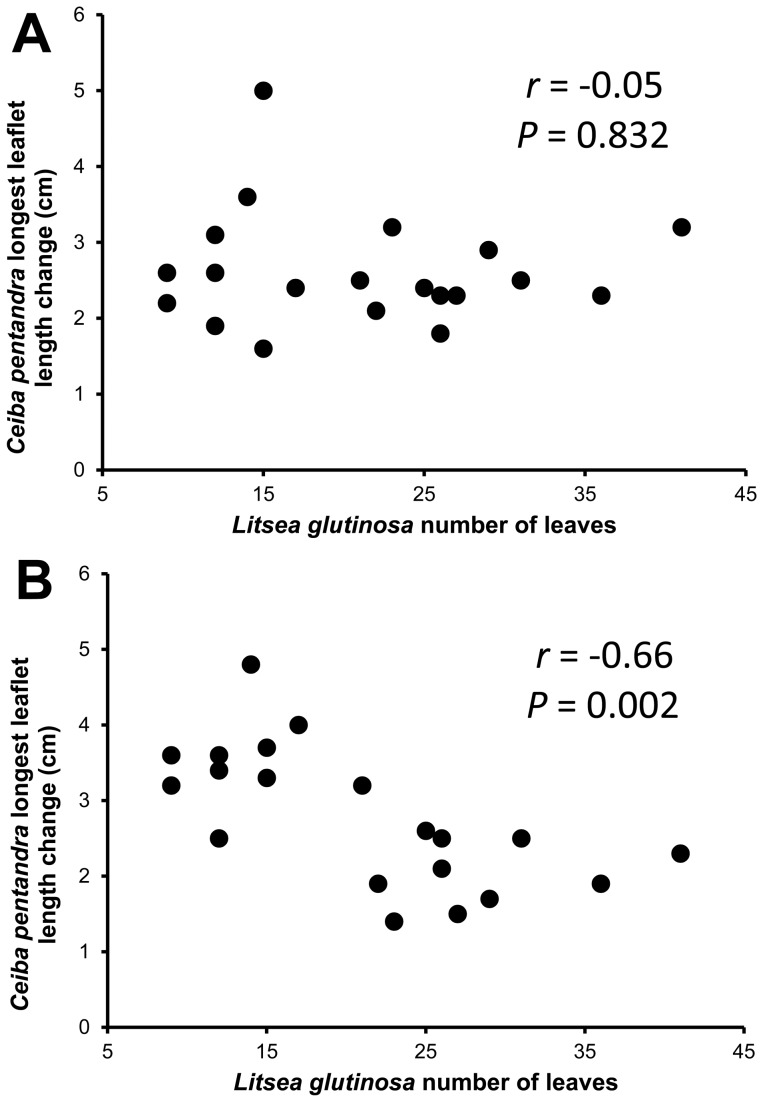
*Ceiba pentandra* growth versus *Litsea glutinosa* nurse plant size. (A) with potential hyphal network interconnections repeatedly severed, and (B) without hypha severing. Longest leaflet length change from initial measurement (cm) for *C. pentandra* is shown versus the number of leaves on *Litsea glutinosa* nurse plants 141 days after transplant (DAT). With hypha severing, there is no significant association, but in the likely presence of common AM networks a significant negative association suggests belowground competitive interactions.

**Figure 6:**
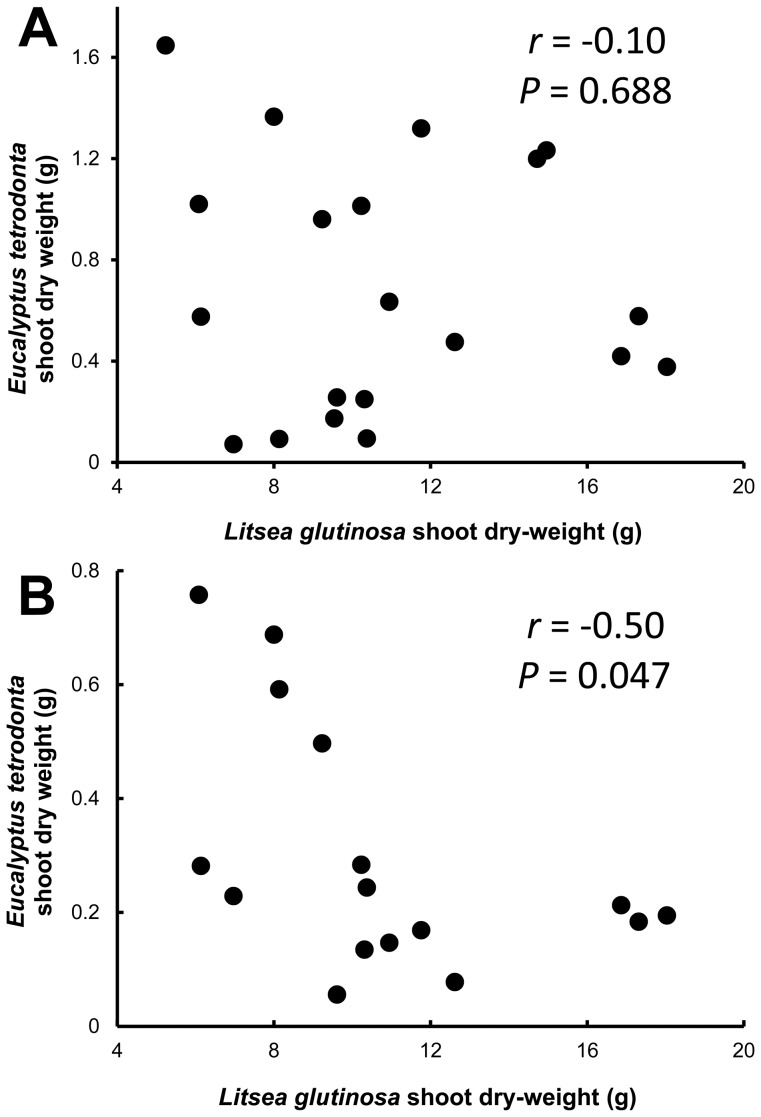
*Eucalyptus tetrodonta* versus *Litsea glutinosa* nurse plant dry weights. (A) with potential hyphal network interconnections repeatedly severed, and (B) without hypha severing. Shoot dry weights (g) of both *E. tetrodonta* and *L. glutinosa* at harvest are shown. With hypha severing, there is no significant association, but in the likely presence of common AM networks a significant negative association suggests belowground competitive interactions.

When we examined associations between *C. pentandra* longest leaflet length change (a proxy for growth rate that was not affected significantly by soil fumigation) and *L. glutinosa* number of leaves on 10 May, 1998 (a proxy for plant size), although there was no significant association for hyphae-severed plants (n = 20, *r* = −0.05, *P* = 0.832; Figure 5A), we found a significant negative association (n = 20, *r* = −0.66, *P* = 0.002; Figure 5B) among networked plants. Similarly, for shoot dry weights of *E. tetrodonta* versus *L. glutinosa* harvested with them, although there was no significant association for hyphae-severed plants (n = 20, *r* = −0.10, *P* = 0.688; Figure 6A), there was a significant negative association among networked plants (n = 16, *r* = −0.50, *P* = 0.047; Figure 6B).

### Iron fertilization of *E. tetrodonta*

**Figure 7:**
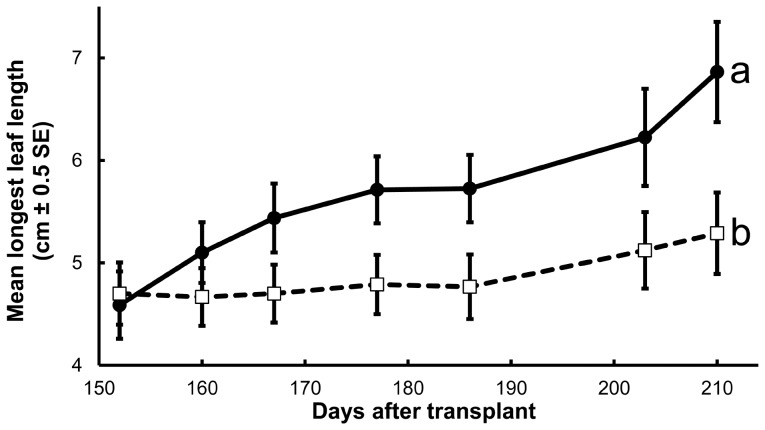
*Eucalyptus tetrodonta* growth response to iron fertilization. Mean longest leaf length (cm±0.5 standard error) is shown from 152 to 210 days after transplant (DAT) to microcosms containing ambient rain forest soil either fertilized with soluble iron (filled circles; solid lines) beginning 130 DAT or not fertilized (open squares; dashed lines). Different lower case letters denote significantly different interactions with time. Soluble iron fertilization improved *E. tetrodonta* longest leaf length increase.

Iron fertilization of *E. tetrodonta* seedlings in ambient soil stimulated growth and remedied chlorosis. Iron fertilization significantly elevated longest leaf length increase (fertilization×time *F*_6,90_ = 4.02, *P* = 0.001; Figure 7), although neither height increase (fertilization×time *F*_6,90_ = 1.87, *P* = 0.095) nor number of leaves increase (fertilization×time *F*_6,90_ = 1.52, *P* = 0.179) was affected significantly. At harvest, mean longest leaf length of iron-fertilized plants exceeded that of not-fertilized plants by 2.0 cm, and iron fertilization had remedied chlorosis in all 5 chlorotic plants among the 8 plants randomly allocated to be fertilized. In contrast, all 4 chlorotic among 9 not-fertilized plants remained chlorotic. Although the two random groups did not differ in the proportion of chlorotic plants before fertilization began (Fisher exact two-tail *P* = 0.637), they differed significantly in the proportion of plants that recovered from chlorosis (Fisher exact two-tail *P* = 0.008). Iron fertilization, however, did not significantly elevate *E. tetrodonta* seedling dry weights (Table 2), nor did hyphae-severed plants differ from networked plants after fertilization (Table S1).

### *E. tetrodonta* foliar mineral nutrient concentrations

After Bonferroni correction, only leaf tissue manganese concentration was affected significantly among the four treatments shown in Table 3. Both hyphae-severed and networked *E. tetrodonta* seedlings grown in fumigated soil (at a mean of 204 mg kg^−1^) had 2.6 times the mean manganese concentration of those in ambient soil. Without Bonferroni correction, boron differed only for fumigated, hyphae-severed versus networked plants (plants in ambient soil are intermediate and cannot be distinguished statistically from either of those groups). Although not significantly different among these four groups (*P* = 0.061), iron had a conspicuously high mean concentration in fumigated soil for networked plants which also had the smallest mean leaf dry weight of all groups (Table 2). In ambient soil, severing hyphae had no significant effects either with or without iron fertilization (Table S2). Among all treatments and eleven elements, the only element that was correlated significantly (negatively) with leaf dry weight was iron (n = 27, *r* = −0.62, *P*<0.001). There were no significant differences among treatments in the total foliar content of any element.

**Table 3:** Leaf element concentrations of *Eucalyptus tetrodonta* seedlings in response to common mycorrhizal network hypha severing in fumigated rain forest soil and to soluble iron fertilization in non-fumigated, ambient soil.

	Fumigated soil (141 DAT)		Ambient soil (210 DAT)		
Element (units)	Hyphae-severed	Networked	No fertilization	Iron fertilization	*F*_3,23_ (KW), *P*
P (%)	0.14 (0.02)	0.26 (0.12)	0.14 (0.03)	0.16 (0.01)	(1.46), 0.692
S (%)	0.14 (0.01)	0.24 (0.08)	0.15 (0.02)	0.14 (0.01)	1.48, 0.246
K (%)	0.54 (0.04)	1.35 (0.62)	0.56 (0.08)	0.55 (0.05)	1.93, 0.153
Ca (%)	1.17 (0.15)	1.26 (0.35)	1.18 (0.14)	0.97 (0.04)	(1.62), 0.655
Mg (%)	0.45 (0.04)	0.48 (0.13)	0.50 (0.05)	0.40 (0.03)	0.34, 0.800
Na (mg kg^−1^)	0.2 (0.02)	0.3 (0.08)	0.4 (0.03)	0.3 (0.02)	(7.28), 0.064
Zn (mg kg^−1^)	57 (7.1)	60 (21.4)	52 (8.9)	43 (2.4)	0.40, 0.755
Cu (mg kg^−1^)	113 (8.6)	116 (32.8)	125 (24.7)	71 (11.1)	1.35, 0.283
Mn (mg kg^−1^)	**204** (17.4)	**204** (62.0)	88 (8.1)	69 (5.4)	5.81, 0.004
Fe (mg kg^−1^)	335 (68.9)	647 (162)	256 (65.0)	317 (94.6)	2.83, 0.061
B (mg kg^−1^)	29 (1.5)	45 (10.6)	39 (2.5)	31 (1.9)	(9.89), 0.020

Values are means±1 standard error in parentheses with significantly highest values after Bonferroni correction (*P*≤0.0046) in bold. All elements with homoscedastic variances were compared by one-way analyses of variance (*F* and *P* shown), but P, Ca, Na, and B, for which variances were heteroscedastic were compared by non-parametric Kruskal-Wallis analyses (KW = Kruskal-Wallis statistics shown in parentheses). In methyl-bromide fumigated soil, n = 9 for hyphae-severed and 6 for networked treatments; in non-fumigated, ambient soil, there were 6 samples in each group. DAT = days after transplant.

## Discussion

Our results strongly support our hypotheses that AM fungi are detrimental to *E. tetrodonta* seedlings, and that the detriment involves iron limitation. The detriment likely is a consequence of two complementary phenomena: common mycorrhizal network interconnections mediate plant competition [33], [41], [42] to the disadvantage of *E. tetrodonta*, and active AM fungus mycelia may lead to iron sequestration whether or not those mycelia interconnect plants. Although our study did not trace hyphal interconnections between plants, negative associations between *C. pentandra* or *E. tetrodonta* and *L. glutinosa* only when hyphae were not severed are consistent with common mycorrhizal networks being present, as is the enhancement of *E. tetrodonta* mean root colonization in fumigated soil when networked [41].

The negative effects of potentially networked hyphae on the rates of increase of all *E. tetrodonta* morphological parameters are somewhat surprising because of the extent to which *E. tetrodonta* seedlings formed AM (seedlings in all treatments except fumigated, hyphae-severed, had 45% mean root length colonized, similar to the 45% mean colonization of *C. pentandra* across all treatments). AM improved *C. pentandra* growth in ambient versus fumigated soil, but in distinct contrast, the growth of *E. tetrodonta* seedlings with the lowest mean root colonization (29%) in hyphae-severed, fumigated soil exceeded growth in all other treatments. Consequently, little-colonized seedlings might be the most likely to establish successfully in the stressful milieu of rain forest gaps. Indeed, under our experiment's conditions that simulated non-burnt rain forest gaps, three networked *E. tetrodonta* seedlings in ambient soil died. Although AM previously have been reported to cause growth depressions of plants grown singly in pots, those negative effects usually have reflected AM failing to repay their cost to the host under conditions of low light, extremely low available phosphorus, or high phosphorus fertilization initiated after abundant mycorrhizas already had formed [43]. None of those effects is likely to explain the suppression of *E. tetrodonta* growth, because under the same conditions in which AM were detrimental to *E. tetrodonta*, AM benefitted *C. pentandra*. Even though mycorrhizas usually are considered to be archetypical mutualisms, our work underscores that mutualism is not constitutive but is context-dependent [44].

It is peculiar that *E. tetrodonta* seedlings sustain AM that might contribute to their deaths in rain forest soil. Natural selection may not favor rejection of AM because AM are beneficial in other contexts or if formed by a different suite of fungus species. For instance, many shrub-layer species in Northern Territory savannas form AM [14], so *E. tetrodonta* seedlings could be connected to common AM networks in savanna. Available iron is two-and-a-half times more abundant there than in rain forest soil, however, perhaps mitigating potential negative effects of AM. Alternatively, if *E. tetrodonta* seedlings quickly form ectomycorrhizas [45]–[47] amidst dominant ectomycorrhizal adults [14], they may avoid AM networks and diminish selection pressure to reject AM. Nevertheless, a price paid for lacking the capacity to reject AM might be inability to invade rain forest.

### *E. tetrodonta* growth enhancement by hypha severing

Our treatments reduced but did not eliminate AM colonization of either host species. Nevertheless, fumigated, hyphae-severed compartments probably had the least AM inoculum of any treatment while, ambient, networked compartments probably had the most inoculum. Reduction of colonization of *C. pentandra* by fumigation even when hyphae were not severed implies that fumigation diminished viable AM fungus propagules to an extent not entirely compensated by hyphae from the nurse plant. Repeated severing did not significantly affect *C. pentandra* root colonization, however, probably because roots of these relatively large plants came sufficiently close to mesh root barriers to be colonized in the 2–3 d interval between hypha severing. Once established, colonization could spread within a root system to reach an asymptote [48]. In contrast, in fumigated soil, repeated hypha severing did retard root colonization of *E. tetrodonta* which was smaller than *C. pentandra*, thereby probably prolonging the time needed for roots to closely approach mesh barriers. Not severing hyphae in fumigated soil compartments, however, elevated AM colonization of *E. tetrodonta* at 141 DAT to a level similar to that at 210 DAT, thereby supporting that seedlings and nurse plants indeed may have been connected by common mycorrhizal networks. No effect of hypha severing at 210 DAT, suggests that *E. tetrodonta* seedlings had attained asymptotic colonization.

In fumigated soil, repeated hypha severing doubled *E. tetrodonta* whole plant dry weight versus that of networked plants. Fumigation, however, neither significantly affected *E. tetrodonta* growth rates (assessed with morphological measurements), nor did fumigation interact significantly with hypha severing. Therefore, any fertilization effect of fumigation such as N and P release from killed microbes [8] or an increase in the ratio of ammonium to nitrate that facilitated iron reduction [49] little affected our results. Furthermore, we failed to detect inhibitory effects on *E. tetrodonta* of non-networked AM. That differs from the pot experiments reported by Stocker [50] and Bowman and Fensham [5] in which non-fumigated rain forest soil was inhibitory to singly-grown eucalypt seedlings. Both those studies' ambient soils, however, had not been maintained plant-free for six months prior to eucalypt planting as had ours. In our microcosms, repeated severing of potential network connections to nurse plants maximized *E. tetrodonta* growth rates only when combined with initial elimination of AM inoculum by fumigation, so it is possible that in ambient soil there may have been an inhibitory effect of non-networked AM that we could not detect statistically.

When hyphae were not severed we found significant negative associations between *C. pentandra* and *L. glutinosa*, and between *E. tetrodonta* and *L. glutinosa* which suggest that belowground competition was mediated across common mycorrhizal networks. The belowground competition was not sufficiently strong to produce a significant beneficial effect of hypha severing for *C. pentandra*, however, because *C. pentandra* were the largest plants and probably the strongest competitors overall. Aboveground, because of close spacing of the completely randomized microcosms, tall *C. pentandra* were as likely to shade adjacent *E. tetrodonta* seedlings as they were to shade their accompanying nurse plants, thereby probably distributing aboveground competition relatively evenly across our entire experiment. Belowground, however, hyphal interconnections likely influenced competition within individual microcosms. Even though root system overlap was prevented by mesh barriers, AM fungus hyphae could cross the mesh and might have redistributed mineral nutrients [20], [30], [51]. Similar to our results, other greenhouse experiments have found that plants interconnected by AM networks can compete strongly belowground [33]–[35], [41], [42].

### Iron deficiency of *E. tetrodonta* in rain forest soil

The response of *E. tetrodonta* seedlings to iron fertilization of ambient soil unambiguously demonstrated that iron was a growth-limiting mineral nutrient. Fertilization not only stimulated leaf growth but also completely eliminated chlorosis. Iron limitation of *E. tetrodonta* in rain forest soil is consistent with only two-fifths as much iron being available in rain forest as in savanna soil. Moreover, iron fertilization has been reported to remedy chlorosis of several eucalypt species [36]. Although the highest mean foliar iron concentration was found for the smallest plants overall in our experiment, that does not contraindicate iron as a growth-limiting mineral nutrient because iron can remain in leaf veins and be physiologically ineffective [49]. Extreme chlorosis that greatly reduces leaf growth can result in exceptionally high iron concentrations [52]–[54], as we found.

*E. tetrodonta* leaf analyses do not suggest that any element other than iron was limiting. Although manganese concentration was highest for all *E. tetrodonta* grown in fumigated soil, iron-fertilized seedlings at 210 DAT attained the highest mean whole plant dry weight with only one-third the manganese concentration of plants in fumigated soil. Furthermore, no analyzed element's total foliar content differed significantly among treatments. Even though we did not analyze nitrogen, it is unlikely that nitrogen limited growth because iron fertilization stimulated growth without supplemental nitrogen.

AM have been reported to improve the iron nutrition of woody plants under some conditions [55]–[57], but improved iron uptake was unlikely in our experiment because the best *E. tetrodonta* growth was associated with the lowest mean AM colonization. Alternatively, under some conditions AM fungi might exacerbate iron deficiency by producing the glycoprotein glomalin [58] which can contain 8.8% iron by weight [59]. If glomalin sequesters iron, then for such an effect to have operated in our experiment, glomalin in fumigated, hyphae-severed compartments would have had to diminish during the six months before *E. tetrodonta* seedlings were planted. Such a rapid decline of immunoreactive, easily-extractable glomalin is supported by findings of Preger et al. [60].

### Fire, mycorrhiza networks, and the rain forest–savanna boundary

If the hypothesized decline of glomalin contributed to *E. tetrodonta* seedling growth in fumigated, hyphae-severed rain forest soil, then intense fires must be capable of leading to similar diminution of glomalin in the field. Knorr et al. [61], however, found no effect of fire on glomalin in Ohio, U.S.A. oak forest soils. Nevertheless, their soils had been stored for 1–7 years at room temperature, and loss of immunoreactivity during storage might have obscured effects of fire. Alternatively, some studies report direct effects of fire on AM fungi, but others report none or only indirect effects through reduced numbers of live host plants [16].

If a severe fire burns into rain forest, microbe death and glomalin decline might elevate iron availability, and AM host death might help *E. tetrodonta* seedlings avoid inclusion in common AM networks that exacerbate belowground competition with rapidly-growing rain forest species. Once ECM fungi invade to associate with establishing seedlings that have survived sufficiently long for the fungi to encounter them [62], ECM fungus siderophores [27], [63] could maintain iron availability, a positive feedback.

ECM networks in savanna may enhance the resistance of savanna to invasion by AM rain forest plants. ECM networks probably have far greater potential for hydraulic redistribution [64] than common AM networks because ECM canopy trees have deep roots [65] and some ECM fungi produce rhizomorphs or mycelial strands. Indeed, the open savanna canopy might elevate ground-level temperature [66] and reduce humidity to levels with which rain forest plant species have difficulty. Nevertheless, in the absence of fire, rain forest species slowly may invade savanna, perhaps by sharing AM fungus associates with AM savanna species [14] and thereby coping with low mineral nutrient availability. If such associations extend common AM networks and elevate glomalin, then phosphorus availability might increase because of iron sequestration [67], further favoring invasion by rain forest species.

Fire–mycorrhiza–vegetation feedbacks likely provide resilience to rain forest and savanna alternative stable-state systems, although rain forest resilience is overcome by the ashbed effect. The mechanisms of that effect are unresolved, however, and in our experiments, most suggested ashbed mechanisms played no role. No ECM [12], [13] formed, nor were pathogen effects [10] apparent (because iron fertilization resulted in seedlings in ambient soil recovering full health). We detected no fertilization effect of soil fumigation that killed microbes [8], but any release of iron–possibly because of glomalin degradation–likely would have been more important than elevation of nitrogen or phosphorus. The most important feature of our experiment for facilitating *E. tetrodonta* growth was disruption of common AM networks, not previously recognized as part of the ashbed effect, but a potential consequence of fire-caused mortality of AM hosts.

Our results sharply distinguish the possible roles of different mycorrhiza types in influencing plant community composition. ECM and AM should not be viewed simply as alternative plant adaptations that minimize niche overlap and foster coexistence of their hosts. We have shown that common AM networks can be actively antagonistic to an eventual ECM host.

## Supporting Information

Table S1 **Dry weight responses of** ***Eucalyptus tetrodonta*** **seedlings to soluble iron fertilization and to common mycorrhizal network hypha severing in ambient rain forest soil 210 days after transplant.**
(DOCX)

Table S2 **Leaf element concentrations of** ***Eucalyptus tetrodonta*** **seedlings in response to soluble iron fertilization and to common mycorrhizal network hypha severing in ambient rain forest soil 210 days after transplant.**
(DOCX)
