# Supplementary material for: Arbuscular-Mycorrhizal Networks Inhibit Eucalyptus tetrodonta Seedlings in Rain Forest Soil Microcosms
Source: PLoS One. 2013 Feb 27;8(2):e57716. doi: 10.1371/journal.pone.0057716 (PMC3584021; doi:10.1371/journal.pone.0057716)
Supplement: Table S1 — Dry weight responses of Eucalyptus tetrodonta seedlings to soluble iron fertilization and to common mycorrhizal network hypha severing in ambient rain forest soil 210 days after transplant. (DOCX) [file pone.0057716.s001.docx]

Table S1. Dry weight responses of *Eucalyptus tetrodonta* seedlings to soluble iron fertilization and to common mycorrhizal network hypha severing in ambient rain forest soil 210 days after transplant.

|  | No fertilization | | | Iron fertilization | | |
| --- | --- | --- | --- | --- | --- | --- |
| Response (units) | Hyphae severed | Networked | DF, *t, P* | Hyphae severed | Networked | DF, *t, P* |
| Leaf weight (g) | 0.513 (0.190) | 0.214 (0.088) | 7, 1.31, 0.231 | 0.494 (0.207) | 0.520 (0.349) | 6, -0.07, 0.948 |
| Stem weight (g) | 0.214 (0.073) | 0.126 (0.039) | 7, 0.99, 0.357 | 0.198 (0.071) | 0.214 (0.121) | 6, -0.12, 0.907 |
| Root weight (g) | 0.321 (0.143) | 0.069 (0.027) | 4.3, 1.75, 0.151 | 0.211 (0.105) | 0.198 (0.161) | 6, 0.07, 0.945 |
| Fine roots:leaf | **0.483** (0.119) | 0.115 (0.022) | 4.3, 3.05, 0.035 | 0.188 (0.037) | 0.170 (0.076) | 6, 0.24, 0.818 |

Values are means ± 1 standard error in parentheses with significantly highest values (without Bonferroni correction) in bold. Degrees of freedom (DF), *t* statistics, and associated two-tail probabilities (*P*) are shown. Satterthwaite’s *t*-test (with fractional degrees of freedom) was used when variances were not homogenous. For both hyphae severed treatments, n = 5; not fertilized, networked n = 4, and iron fertilized, networked n = 3.
