# Supplementary material for: Arbuscular-Mycorrhizal Networks Inhibit Eucalyptus tetrodonta Seedlings in Rain Forest Soil Microcosms
Source: PLoS One. 2013 Feb 27;8(2):e57716. doi: 10.1371/journal.pone.0057716 (PMC3584021; doi:10.1371/journal.pone.0057716)
Supplement: Table S2 — Leaf element concentrations of Eucalyptus tetrodonta seedlings in response to soluble iron fertilization and to common mycorrhizal network hypha severing in ambient rain forest soil 210 days after transplant. (DOCX) [file pone.0057716.s002.docx]

Table S2. Leaf element concentrations of *Eucalyptus tetrodonta* seedlings in response to soluble iron fertilization and to common mycorrhizal network hypha severing in ambient rain forest soil 210 days after transplant.

|  | No fertilization | | Iron fertilization | |  |
| --- | --- | --- | --- | --- | --- |
| Element (units) | Hyphae-severed | Networked | Hyphae-severed | Networked | *F*_3,7_ (KW), P |
| P (%) | 0.17 (0.03) | 0.13 (0.04) | 0.15 (0.03) | 0.18 (0.04) | 0.33, 0.802 |
| S (%) | 0.12 (0.02) | 0.20 (0.03) | 0.15 (0.02) | 0.13 (0.03) | 1.93, 0.214 |
| K (%) | 0.57 (0.10) | 0.65 (0.12) | 0.53 (0.08) | 0.59 (0.12) | 0.23, 0.876 |
| Ca (%) | 1.19 (0.16) | 1.31 (0.19) | 1.00 (0.14) | 0.93 (0.19) | (1.67), 0.644 |
| Mg (%) | 0.41 (0.05) | 0.59 (0.07) | 0.43 (0.05) | 0.35 (0.07) | (4.62), 0.202 |
| Na (mg kg^-1^) | 0.3 (0.05) | 0.4 (0.08) | 0.3 (0.04) | 0.3 (0.06) | (1.81), 0.613 |
| Zn (mg kg^-1^) | 38 (5.7) | 77 (7.0) | 44 (4.9) | 41 (7.0) | (4.82), 0.186 |
| Cu (mg kg^-1^) | 81 (22) | 169 (27) | 74 (19) | 66 (27) | (4.24), 0.237 |
| Mn (mg kg^-1^) | 75 (7.8) | 108 (9.6) | 74 (6.8) | 60 (9.6) | (6.00), 0.112 |
| Fe (mg kg^-1^) | 131 (112) | 340 (137) | 270 (97) | 412 (137) | (3.59), 0.309 |
| B (mg kg^-1^) | 34^ab^ (2.4) | 46^a^ (3.0) | 32^b^ (2.1) | 31^b^ (3.0) | 5.91, 0.025 |

Values are means ± 1 standard error in parentheses. Elements with homoscedastic variances were compared by one-way analysis of variance (F and P shown), but those for which variances were heteroscedastic were compared by non-parametric Kruskal-Wallis analyses (KW = Kruskal-Wallis statistics shown in parentheses). Although Boron values would not differ significantly after Bonferroni correction, without correction those followed by the same superscript letter do not differ significantly by Tukey’s HSD test. For both networked treatments, n = 2; not fertilized, hyphae severed n = 3, and iron fertilized, hyphae severed n = 4.
